# Current status of intestinal parasitic infections and associated factors among primary school children in Birbir town, Southern Ethiopia

**DOI:** 10.1186/s12879-019-3879-5

**Published:** 2019-03-19

**Authors:** Getaneh Alemu, Ashenafi Abossie, Zerihun Yohannes

**Affiliations:** 1 Department of Medical Laboratory Science, Bahir Dar University, Bahir Dar, Ethiopia; 2 Department of Medical Laboratory Science, Arba Minch University, Arba Minch, Ethiopia

**Keywords:** School age children, Intestinal parasitic infection, Prevalence

## Abstract

**Background:**

Intestinal parasites still pose major public health problems in developing countries like Ethiopia. Local epidemiological data is indispensable in order to design and monitor prevention and control strategies. Therefore the present study aimed to assess the prevalence of intestinal parasitic infections and associated factors among students at Birbir town, Southern Ethiopia.

**Methods:**

A school-based cross-sectional study was conducted from March to May 2018. Three hundred fifty-one students, who were selected by stratified followed by systematic random sampling, participated in the study. Socio-demographic information was collected using a structured questionnaire. Anthropometric measurements of height and weight were taken at the time of interview. Stool samples were collected and processed by direct wet mount and formol-ether concentration techniques for microscopic detection of intestinal parasites. Data was analyzed using SPSS version 20.

**Results:**

Among 351 (180 male and 171 female) children participated, 135 (38.5%) and 216 (61.5%) were within the age groups of 5–9 and 10–14 respectively. Ninety five (27.1%; 95%CI: 22.2–31.9) of them were tested positive for intestinal parasites. Helminths and protozoa account 21.1 and 7.1% prevalences respectively. Seventy eight children were infected with a single parasite species while 17 were positive for double or triple infections. *A. lumbricoides* (31, 8.8%) was the most frequently detected parasite followed by *T. trichiura* (20, 5.7%) and hookworms (19, 5.4%). Age group of 10–14 years (AOR = 2.51; 95%CI: 1.41–4.45, *p* = 0.002) and absence of hand washing habit after toilet (AOR = 4.49; 95%CI: 2.00–10.1, *p* = 0.001) were significantly associated with intestinal parasitic infection.

**Conclusions:**

The prevalence of intestinal parasitic infection among school age children is still unacceptably high. Age group of 10–14 year old and not having habit of hand washing after toilet were significantly associated with intestinal parasitic infection. The ongoing school based deworming should be strengthened and be integrated with school health programs.

**Electronic supplementary material:**

The online version of this article (10.1186/s12879-019-3879-5) contains supplementary material, which is available to authorized users.

## Background

Intestinal parasitic infections (IPI) still pose one of the major public health problems that about 3.5 billion people are infected globally [1]. The majority of parasite associated scourge is concentrated in the developing, mainly sub-saharan, countries due to inadequate water supply, poor environmental sanitation, fast population growth, and other economic and social problems [2]. It is estimated that more than 10.5 million new cases are reported annually and *Ascaris(A) lumbricoides*, hookworms, *Trichuris(T) trichiura*, *Giardia(G) lamblia*, *Entamoeba (E) histolytica* and *Schistosoma* species are the most common intestinal parasites [3].

In developing regions, particularly Africa, more than 173 million people are infected with *A. lumbricoides* while 198 million and 162 million people are infected with hookworms and *T. trichiura* respectively [4]. In Ethiopia 81 million people live in endemic areas, of which 25.3 million are school-age children (SAC) [5, 6].

As studies indicated that soil transmitted helminths (STH) such as *A. lumbricoides, T. trichuria* and hookworms have been associated with growth retardation and impairment in cognitive development, anemia and vitamin A deficiency [7, 8]. Annually millions of people infected with *G.lamblia* manifest diarrhea, malnutrition and wasting [9]. Similarly, *E. histolytica* causes intestinal and extra-intestinal amoebiasis in large number of people resulting in many deaths annually [10]. Children are more vulnerable to serious complications of parasitic infection such as malnutrition, anemia, bowel obstruction, and learning disabilities as compared to adults [11].

School children are the most commonly affected groups due to their typical hand-mouth activity, uncontrolled fecal activity, water contamination and their immature immune system. Their behavioral patterns are also associated with the high prevalence of IPI compared to adults [12].

Parasitic infections are more prevalent in populations with low-household income, poor practices of personal hygiene and environmental sanitation, over-crowded living conditions, and limited access to safe water supply [9]. Access to basic sanitation facilities in Ethiopia, especially in rural areas, is quite poor.

According to the Ethiopian Demography and Health Survey report of the year 2016, 97 and 57% of urban and rural households have access to an improved source of drinking water respectively. Only 60% of households had hand washing facility at home. One in three households has no toilet facility (39% in rural areas and 7% in urban areas) [13]. Poor knowledge, attitudes, and practices about transmission cycle and health impact of parasites are among the risk factors for developing IPI [14]. Under-nutrition and parasitic diseases have similar geographical distribution with the same people experiencing both for much of their lives [15]. Moreover, IPI are thought to contribute to under-nutrition through reduction in digestion and absorption, bowl obstruction and loss of nutrients. On the other side, under-nutrition can make children more susceptible to parasitic infections [16].

Prevalence of IPI was assessed in Mirab Abaya district focusing on hookworms in 2013. This previous study failed to assess the prevalence of intestinal protozoa [17]. Moreover, national school based deworming has been implemented since 2015 and efforts have been made in order to improve Water, Sanitation and Hygiene (WASH). Therefore periodic monitoring of the effectiveness of such control activities at different geographical contexts benefits the concerned health sector and stakeholders. Hence the aim of the present study was to assess prevalence of IPI and associated factors among school children attending primary school in Birbir town.

## Methods

### Study design and area

A cross sectional study was conducted from March to June 2018 among school children attending primary school in Birbir town. Mirab abaya is one of the woredas of Gamo Gofa zone in the Southern part of Ethiopia. It is located about 45 Kms north of Arbaminch town, 236 Kms from Hawassa and 465 kms from Addis Ababa and its average temperature is 36^0^c with total annual rain fall ranging from 580 mm to 1100 mm [18]. The climate is conducive for survival of many of human parasites. There is one full cycle primary school in the town which we include in the present study.

### Sample size and sampling technique

A single population proportion formula was used to estimate the sample size (n). Taking 27.7% prevalence (p) of intestinal parasitosis from previous similar study [19], 95% confidence level (Z = 1.96) and 5% margin of error (d = 0.05), the initial sample size was 308.

$$ \mathrm{n}=\frac{\mathrm{Z}{\left(\upalpha /2\right)}^{2\ast}\mathrm{P}\left(1-\mathrm{P}\right)}{{\mathrm{d}}^2}=\frac{(1.96)^{2\ast }(0.277)\left(1-0.277\right)}{(0.05)^2}=308 $$

By considering a 15% (47) non response rate, the final sample size was 355. The total sample size was proportionally allocated to the grades and classes. Finally, participant children from each class were selected using systematic random sampling technique by using class rosters as a sampling frame. The inclusion criteria were being: with in the age group of 5–14 years old; permanent resident in the study area at least for the previous 1 month before data collection; volunteer (both children and parents) to participate in the study. Children who took anti helminthic or anti protozoa drugs within the last 3 months and those who are unable to respond to research questions due to any disability were excluded.

### Data collection

#### Socio-demographic data

A structured questionnaire was used to collect data from children. Questionnaire involves information on socio-demographic, environmental, and sanitary facilities of students. Data regarding water, hygiene and sanitation of the school was collected by interview with school director and direct observation (Additional file 1).

#### Anthropometry measurements

Weight and height of each child were measured for the assessment of children’s nutritional status.

Weight was measured using digital weight scale. Height was measured using the vertical measuring tape by sticking on the rod. The children’s shoes, hair clips, and braids were removed before measurement; children positioned feet together flat on the ground, heels touching the back plate of the measuring instrument, legs straight, buttocks and scapula against the backboard; a ruler was used to straighten the hair and determine the height. Body-mass-index (BMI), calculated from weight and height, was used as an indicator of the nutritional status of children. Children with BMI < 18.5 were grouped as malnourished [20].

#### Stool examination

Polyethylene screw cupped stool container was given to each student to bring stool specimen following the interview. Direct saline and iodine wet mount preparations were examined within 30 min after collection. Temporary microscopy station was set in the school compound for this purpose. The remaining sample was preserved with 10% formalin and transported to Birbir health center laboratory. Then all transported samples were processed and examined using the formol-ether concentration technique following standard protocol explained elsewhere [21]. Pre-test was conducted in Sikela primary school (located in Arba Minch town) by recruiting 18 students (5% of the total sample size) to assess problems related to questionnaires and sample collection and processing. Problems identified during pre-test were corrected before the start of actual data collection.

### Statistical analysis

Data were edited, cleaned, entered and analyzed using SPSS version 20.0. Descriptive statistics were calculated to describe the study population characteristics. Bivariate logistic regression was used to assess associations between independent and outcome variables. Multivariate regression model then followed for variables with *p* ≤ 0.25 in the bivariate analysis. Association between variables was considered statistically significant only if *p*-value ≤0.05 at 95% confidence level. Compiled results were presented in text and tables.

## Results

### Water, sanitation and hygiene facilities in the school

The main water source in the school was pipe water. It was functional only for 2–4 days per week and does not fulfill the needs of the school. There were no drinking water facilities accessible to students with physical disabilities. The school has two toilets (one for male and the other for female) for students and one separate toilet for teachers. The students’ toilet has two rooms and each room has four compartments. All three toilets lack urinals and hand washing facilities. There were no toilets accessible to students with physical disabilities, but toilets available in the school were well designed for younger students. The school has only one hand washing station, but there was no soap available during the visit.

### Socio- demographic characteristics of study participants

Among a total of 355 students participated, data from 351 children were complete for analysis (51.3% male and 48.7%) female. One hundred thirty five (38.5%) and 216 (61.5%) participants were within the age groups of 5–9 and 10–14 years old respectively. Majority of the participants (98%) responded as they wash their hands before meal. Similarly 318 (90.6%) children had habit of hand washing after toilet. Three hundred twenty five (92.6%) of them frequently wear shoe and 334 (95.2%) had latrine at home (Table 1).

**Table 1:** Socio-demographic characteristics of primary school children (*n* = 351) at Birbir town, Southern Ethiopia, 2018

Variables	Category	Frequency (%)
Age group (years)	5–9	135 (38.5)
	10–14	216 (61.5)
Gender	Male	180 (51.3)
	Female	171 (48.7)
Residence	Urban	247 (70.4)
	Rural	104 (29.6)
Grade level	1–4	234 (66.5)
	5–8	117 (33.5)
Education level of caregiver	Unable to read right	111 (31.6)
	Primary	186 (53)
	Secondary and above	54 (15.4)
Family size	≤5	199 (56.7)
	> 5	152 (43.3)
Habit of eating fruit or Vegetables	Yes	346 (98.6)
	No	5 (1.4)
Wash fruits and vegetables before eating	Yes	303 (86.3)
	No	48 (13.7)
Habit of hand washing before meal	Yes	344 (98)
	No	7 (2.0)
Habit of hand washing after soil contact	Yes	289 (82.3)
	No	62 (17.7)
Habit of hand washing after toilet	Yes	318 (90.6)
	No	33 (9.4)
Source of water for washing clothes and utensils	Piped water	311 (88.6)
	River/Lake	40 (11.4)
Source of water for bathing	Piped water	308 (87.7)
	River/Lake	43 (12.3)
Source of water for drinking	Piped water	321 (91.5)
	River/Lake	30 (8.4)
Habit of swimming	Yes	122 (34.8)
	No	229 (65.2)
Habit of shoe wearing	Yes	325 (92.6)
	No	26 (7.4)
Have latrine at home	Yes	334 (95.2)
	No	17 (4.8)
BMI	< 18.5	73 (20.8)
	≥18.5	278 (79.2)

### Prevalence of intestinal parasites

Of the total 351 stool samples examined, 95 (27.1%; 95%CI: 22.2–31.9) were positive at least for one intestinal parasite. Seventy eight children were infected by single parasite species while 17 (4.8%) children were positive for double or triple infections. Co-infection with *A. lumbricoides* and *T. trichiura* was most common observed in five children. The prevalence of helminths alone was 21.1% (95%CI: 16.8–25.6) and seven intestinal helminth species were detected; *A. lumbricoides* (31, 8.8%) being the most frequent followed by *T. trichiura* (20, 5.7%) and hookworms (19, 5.4%). Twenty five (7.1%; 95%CI: 4.6–10.0) children were infected by protozoan parasites. Two intestinal protozoa namely *G. lamblia* (4.8%; 95%CI: 2.8–7.1) and *E. histolytica/dispar* (2.6%; 95%CI: 1.1–4.3) were detected (Table 2).

**Table 2:** Prevalence of intestinal parasites among primary school children (*n* = 351) at Birbir town, Southern Ethiopia, 2018

Parasites	Frequency	Percentage
*Ascaris lumbricoides*	31	8.8
Hookworms	19	5.4
*Hymenolepis nana*	6	1.7
*Schistosoma mansoni*	4	1.1
*Trichuris trichuria*	20	5.7
*Strongyloides stercoralis*	7	2.0
*Entrobius vermicularis*	2	0.6
*Giardia lamblia*	17	4.8
*Entamoeba histolytica/dispar*	9	2.6

### Factors associated with intestinal parasitic infection

Age group has significant association with IPI that children who are within the age group of 10–14 years old were 2.5 times more likely to acquire intestinal parasites compared to 5–9 years old children (AOR = 2.506, 95% CI: 1.411–4.451, *p* = 0.002). Habit of hand washing after toilet was also significantly associated with IPI (*p* = 0.001). Children who do not wash their hands after toilet were almost 4.5 times at higher risk of infection compared to those having habit of frequent hand washing after toilet (AOR = 4.487, 95% CI: 1.998–10.074). All other considered factors were not associated with IPI (Table 3).

**Table 3:** Factors associated with intestinal parasitic infection among primary school children (*n* = 351) at Birbir town, Southern Ethiopia, 2018

Variables	Category	Number examined	Rate of parasite infection N (%)	COR (95%CI)	*P*-value	AOR (95%CI)	*P*-value
Age group (in years)	5–9	135	21(15.5)	1		1	
	10–14	216	74(34.3)	2.785(1.617–4.796)	0.001	2.506(1.411–4.451)	0.002
Gender	Male	180	47(26.1)	1.104(0.689–1.769)	0.680		
	Female	171	48(28.1)	1			
Residence	Urban	247	56(22.7)	1		1	
	Rural	104	39(37.5)	2.046(1.246–3.362)	0.005	1.397(0.737–2.647)	0.305
Grade level	1–4	173	61(35.3)	1			
	5–8	117	34(29.1)	0.861(0.525–1.441)	0.552		
Educational level of care giver	Unable to read and write	111	29(26.1)	1.191(0.579–2.449)	0.636		
	Primary	186	50(26.9)	1.145(0.587–2.234)	0.691		
	Secondary and above	54	16(29.6)	1			
Family size	≤5	190	38(20.0)	1		1	
	> 5	161	55(34.2)	2.065(1.279–3.333)	0.003	1.498(0.880–2.551)	0.137
Wash fruits and vegetables before consumption	Yes	303	78(25.7)	1			
	No	48	15(31.3)	1.267(0.654–2.455)	0.483		
Habit of swimming	Yes	122	36(29.5)	0.829(0.508–1.352)	0.452		
	No	229	57(24.9)	1			
Habit of hand washing after toilet	Yes	318	74(23.3)	1		1	
	No	33	21(63.6)	5.77(2.711–12.282)	0.001	4.487(1.998–10.074)	0.001
Habit of hand washing after soil contact	Yes	289	78(30.0)	1			
	No	62	15(24.2)	1.158(0.613–2.189)	0.651		
Hand washing habit before meal	Yes	344	89(25.9)	1		1	
	No	7	4(57.1)	0.262(0.057–1.192)	0.083	4.366(0.789–14.176)	0.091
Habit of wearing shoe	Yes	325	84(25.8)	1			
	No	26	9(34.6)	0.658(0.283–1.533)	0.332		
Latrine availability	Yes	334	86(25.7)	1		1	
	No	17	7(41.2)	0.495(0.183–1.342)	0.167	1.108(0.291–4.223)	0.880
Source of drinking water	Piped water	321	80(24.9)	1		1	
	River/Lake	30	13(43.3)	0.434(0.202–0.933)	0.033	1.579(0.555–4.492)	0.392
Source of water for bathing	Piped water	308	75(24.4)	1		1	
	River/Lake	43	18(41.9)	0.447(0.231–0.864)	0.017	1.121(0.208–6.032)	0.894
Source of water for washing clothes and utensils	Piped water	311	76(24.4)	1		1	
	River /Lake	40	17(42.5)	0.438(0.222–0.862)	0.017	0.973(0.177–5.354)	0.975
BMI	< 18.5	73	18(24.7)	1.129(0.623–2.046)	0.689		
	≥18.5	278	75(27.0)	1			

*AOR* Adjusted Odd Ratio, *CI* Confidence Interval, *COR* Crude Odd Ratio

## Discussion

The rate of IPI in the present study was 27.1% which goes in line with findings from Ethiopian Somali (26.6%) [22]. Higher prevalence of 35.44% in Homesha (Benishangul); 65.5% in Bahir dar; and 84.3% in Debre Elias (East Gojjam) were reported previously [23–25]. Data for these three studies was collected between 2013 and 2016 which is before or early after the initiation of the Ethiopian national mass drug administration program launched in 2015 for STH and Schistosomiasis. Variations in spatial distribution of intestinal parasites might also confer the difference.

The prevalence of intestinal helminths, excluding protozoa, was 21.1% which is comparable with previous study from the same area (21.8%) [17]. It is expected that the currently ongoing biannual school based deworming and WASH programs would have significantly decreased the magnitude of intestinal helminthiasis. However, because of poor performance in the prevention activities which aids re-infection to occur in dewormed children, the burden of helminths is not acceptably decreasing. A comparative study conducted in Chencha, Ethiopia by Zerihun et al, before and 3 months after deworming indicated almost similar prevalence before and after the trearment (36.8% vs 39.4%), pronouncing the frequency of re-infection [26]. The prevalence of helminths in the present study was lower compared to previous study results from Ethiopia; Arba Minch Zuria district (46.3%), Jiga (58.3%), Wolayta (72.2%) and Chencha (81.0%) [27–30]. The big three STH namely *A. lumbricoides* (8.8%), *T. trichiura* (5.7%) and hookworms (5.4%) were the frequently detected helminths in the present study. This goes in line with the national prevalence and many other previous study reports [17, 19, 21–30].

The prevalence of intestinal protozoa parasites was also considerable in the present study which was 7.1%. Only *G. lamblia* and *E. histolytica/dispar* were detected that the prevalence would be much higher if intestinal coccidians were assessed. *G. lamblia* (4.8%) was more common compared to *E. histolytica/dispar* (2.6%). Lower prevalence of giardiasis (1.4–2%) were reported at different geographical settings of Ethiopia [22, 25] while higher infection rate of 11.4 and 12.65% were reported from Bahir dar and Homesha, Ethiopia [23, 24]. Accessibility of safe water and variations in WASH implementation might bring such differences in addition to other environmental and climatic factors. Similarly many previous studies have indicated higher magnitude of *E. histolytica/dispar* ranging from 6.7 to 14.17% [22–25] with the similar reasons mentioned in the case of giardiasis.

Among all study participants, 17 (4.8%) acquired two parasites or more. This is lower as compared to previous findings from Ethiopia. Results from Arba Minch Zuria district, Homesha, Bahir Dar, Zegie and Hawassa reveal 6.1, 6.3, 16.2, 25 and 25.7% of the study participants were infected at least with two intestinal parasites respectively [23, 24, 27, 31, 32]. Variations in the level of environmental contamination and socioeconomic factors might be responsible for the difference in prevalence of multiple parasitic infections at a time [24]. However the present result was higher compared to findings before 4 years in the same area (2.4%). Variations in the laboratory technique used might be an important factor here among other unforeseen reasons. Stool samples were processed only by Kato katz technique in the previous study; but in the present study, we have examined direct saline wet mount smear (in addition to formol-ether concentration) in order to detect motile trophozoites.

According to the present study, age group of 10–14 year old and not having habit of hand washing after toilet were significantly associated with IPI. SAC with age group of 10–14 years old are said to have increased frequency of environmental exposure (soil, water) compared to younger children. Moreover, children will be engaged in agricultural activities at this age. Hence children of age 10–14 were 2.5 times at higher risk of acquiring IPI compared to those 5–9 years old. Many previous studies failed to show this difference, might be, due to other confounding factors [17, 23, 24, 27]. Many of the intestinal parasites are transmitted via the feco-oral route. As a result, family members, who share toilet, are usually primary sources of infection. In order to avoid such transmissions, frequent and proper hand washing after toilet is strongly recommended. In the present study, children who do not wash their hands after toilet were almost 4.5 times more likely to be infected. Similar association was reported from Homesha district that children who lack hand washing habit before meal and after defecation were 5.45 times at higher risk [23].

Recent studies at different geographical settings of Ethiopia indicated that shoe wearing habit [17, 23–25], consumption of raw/unwashed fruits and vegetables [23, 27], habit of swimming [27], family size [24], cleanness of finger nail and trimming [23, 24] and waste disposal habit [23] were significantly associated with IPI. However, these factors were not associated with IPI and some factors were not assessed in the present study. Hookworms are expected to be associated with shoe wearing habit as the transmission is via skin penetration. However, it is not associated in the present study with possible reason of frequent contact with soil regardless of shoe wearing habit. Because the area is located at low altitude with average annual temperature of 36^0^c [18], children usually wear shorts (exposing their legs). Hence they have direct leg to soil contact when they play and sit. Washing of fruits and vegetables before consumption reduces the risk of acquiring parasite infection [33]. In the present study, majority of children (86.3%) wash fruits and vegetables just before consumption; this might bring non-significant association with IPI. The school is located near Lake Abaya where majority of the students (92.1%) responded as they wash their legs and hands at least once a week (data not shown). This frequent exposure regardless of swimming habit might be responsible for the absence of significant association between habit of swimming and schistosomiasis. Overcrowding or communal living condition is a risk for transmission of some intestinal protozoa (*E. histolytica and G. lamblia cysts*) and helminths (*Enterobius vermicularis* and *Hymenolepis nana*) [34]. Hence these parasites can be transmitted across family members. However, data for the present study was collected at school that students have frequent contact with their class children in addition to their family. Hence, family size alone might not have significant role for the transmission of intestinal parasites. Cleanness of finger nail and trimming, and waste disposal habit were not assessed in the present study.

## Conclusion

The prevalence of IPI among SAC is still unacceptably high. Age group of 10–14 years old and not having habit of hand washing after toilet were strongly associated with IPI. The Federal Ministry of health has planned to control schistosomiasis and STH to a level where it is no longer a public health problem by 2020 [6]. However, the high prevalence of intestinal parasites in the present study alarms the ministry that more effort is needed in order to reach to the goal in the remaining 2 years. Consistent implementation of WASH program in integration with deworming seems indispensable strategy. Implementation of school health program should also be promoted.

## Additional file

Additional file 1: Questionnaire for assessment of Current Status of Intestinal Parasitic Infections and Associated Factors among Students at Birbir Primary School, Southern Ethiopia. (DOCX 20 kb)

## Abbreviations

BMI: Body-Mass-Index
IPI: Intestinal parasitic infections
SAC: School-Age Children
STH: Soil Transmitted Helminths
WASH: Water Hygiene and Sanitation
