# Supplementary material for: Current status of intestinal parasitic infections and associated factors among primary school children in Birbir town, Southern Ethiopia
Source: BMC Infect Dis. 2019 Mar 19;19:270. doi: 10.1186/s12879-019-3879-5 (PMC6425597; doi:10.1186/s12879-019-3879-5)
Supplement: Supplementary file 1 — Questionnaire for assessment of Current Status of Intestinal Parasitic Infections and Associated Factors among Students at Birbir Primary School, Southern Ethiopia. (DOCX 20 kb) [file 12879_2019_3879_MOESM1_ESM.docx]

| **Questionnaire for assessment of Current Status of Intestinal Parasitic Infections and Associated Factors among Students at Birbir Primary School, Southern Ethiopia.** | | | | |
| --- | --- | --- | --- | --- |
| **No.** | **Question** | **Answer** | **Code** | **Skip to** |
| 001 | Age of the child |  | _ |  |
| 002 | Sex of the child | Male  Female | 1  2 |  |
| 003 | Residence | Urban  Rural | 1  2 |  |
| 004 | Grade level | 1-4  5-8  Other | 1  2  3 |  |
| 005 | Educational level of the care giver | Unable to read and write  Primary  Secondary /above | 1  2  3 |  |
| 006 | How many family members are there in your house? |  | | |
| 007 | Where do you get your food? | Cooked at home  From hotel  Some times at home some times in hotel  Other | 1  2  3  4 |  |
| 008 | Do you have habit of eating raw fruits/vegetables? | Yes  No | 1  2 |  |
| 009 | Do you wash fruits and vegetables before eating in raw? | Yes  No | 1  2 |  |
| 010 | Do you have hand washing habit after soil contact? | Yes  No | 1  2 |  |
| 011 | Do you have hand washing habit before eating? | Yes  No | 1  2 |  |
| 012 | Do you have hand washing habit after toilet? | Yes  No | 1  2 |  |
| 013 | Do you have hand washing habit after touching toilet materials? | Yes  No | 1  2 |  |
| 014 | Where do you get water for washing your clothes and house utensils? | Pipe water  River/Lake | 1  2 |  |
| 015 | Where do you get water for bathing? | Pipe water  River /lake | 1  2 |  |
| 016 | Where do you get water for drinking? | Pipe water  River/Lake | 1  2 |  |
| 017 | Do you have swimming habit? | Yes  No | 1  2 |  |
| 018 | Do you have shoe wearing habit? | Yes  No | 1  2 |  |
| 019 | Do you have latrine at home? | Yes  No | 1  2 |  |

**School Observation Check List for assessment of WASH**

| **No** | **Questions** | **Responses** | | **Code** | **Remark** | |
| --- | --- | --- | --- | --- | --- | --- |
| **Water** | | | | | | |
| 027 | What is the school’s *main water source*? | Piped water in to school building | | 1 |  | |
|  |  | Protected dug well | | 2 |  | |
|  |  | Unprotected dug well | | 3 |  | |
|  |  | Protected spring | | 4 |  | |
|  |  | Unprotected spring | | 5 |  | |
| 028 | How often is the water source functional? (check one) | 5-7 days per week  2-4 days per week  Less than 2 days per week | | 1  2  3 |  | |
| 029 | Does it provide enough water for the needs of the school, including water for drinking, hand washing and food preparation? (check one) | Yes  No  The water is not functional | | 1  2  3 |  | |
| 030 | Do you treat water from the source you use at school in any way to make it safer to drink? (check one) | Always  Sometimes  Never | | 1  2  3 |  | |
| 031 | Do children bring their own drinking water from home? (check one) | Most children bring water from home  Some children bring water from home  No children bring water from home | | 1  2  3 |  | |
| 032 | Are drinking water facilities accessible to children with physical disabilities? (check one) | Yes  No | | 1  2 |  | |
| 033 | Can the youngest children in the school get drinking water by themselves (check one)? | Yes  No | | 1  2 |  | |
| **Sanitation** | | | | | | |
| 033 | Does the school have any toilet facilities? (check one; a toilet can be a pit latrine, an improved pit latrine, a flush toilet, a pour-flush toilet, or a composting toilet) | Yes  No | | 1  2 |  | |
| 034 | How many toilet compartments are there in the school for children: (insert number) |  | |  |  | |
| 035 | Does the school also have urinals? (check one) | Yes  No | | 1  2 |  | |
| 036 | Do teachers have their own toilet facilities (separate from children’s facilities)? (check one) | Yes  No | | 1  2 |  | |
| 037 | Are toilets accessible to children with physical disabilities? (check one) | Yes  No | | 1  2 |  | |
| 038 | Are some toilets available in the school designed for younger children? (check one) | Yes  No | | 1  2 |  | |
| **Hygiene** | | | | | | |
| 039 | Does the school have hand washing facilities? (check one) | Yes  No | | 1  2 |  | |
| 040 | How many hand washing stations are there in the school: (insert number) |  | |  |  | |
| 041 | Is sufficient soap (or ash) available? (check one) | Aways  Sometimes  Never | | 1  2  3 |  | |
| 042 | Is hygiene taught in the school? (check one) | Yes  No | | 1  2 |  | |
| 043 | Are hand washing facilities accessible to children with physical disabilities? (Children? (check one) | | Yes all facilities are accessible  Some are  None are | 1  2  3 | |  |
| 044 | Are hand washing facilities accessible to younger children? (check one) | | Yes all facilities are accessible  Some are  None are | 1  2  3 | |  |
